# Engineering of Chinese hamster ovary cells for co-overexpressing MYC and XBP1s increased cell proliferation and recombinant EPO production

**DOI:** 10.1038/s41598-023-28622-z

**Published:** 2023-01-27

**Authors:** Yesenia Latorre, Mauro Torres, Mauricio Vergara, Julio Berrios, Maria Molina Sampayo, Natasha Gödecke, Dagmar Wirth, Hansjörg Hauser, Alan J. Dickson, Claudia Altamirano

**Affiliations:** 1 Escuela de Ingeniería Bioquímica, Pontificia Universidad Católica de Valparaíso, Av. Brasil 2085, Valparaíso, Chile; 2 Faculty of Science and Engineering, Manchester Institute of Biotechnology, University of Manchester, Manchester, UK; 3 Biochemical and Bioprocess Engineering Group, Department of Chemical Engineering, University of Manchester, Manchester, UK; 4 Centro de InmunoBiotecnología, Universidad de Chile, Santiago, Chile; 5 Helmholtz Centre for Infection Research, 38124 Braunschweig, Germany; 6 Centro Regional de Estudio en Alimentos Saludables, R17A10001, Av. Universidad 330, Valparaíso, Chile

**Keywords:** Animal biotechnology, Expression systems, Metabolic engineering

## Abstract

Improving the cellular capacity of Chinese hamster ovary (CHO) cells to produce large amounts of therapeutic proteins remains a major challenge for the biopharmaceutical industry. In previous studies, we observed strong correlations between the performance of CHO cells and expression of two transcription factors (TFs), MYC and XBP1s. Here, we have evaluated the effective of overexpression of these two TFs on CHO cell productivity. To address this goal, we generated an EPO-producing cell line (CHO_EPO_) using a targeted integration approach, and subsequently engineered it to co-overexpress MYC and XBP1s (a cell line referred to as CHOCX_EPO_). Cells overexpressing MYC and XBP1s increased simultaneously viable cell densities and EPO production, leading to an enhanced overall performance in cultures. These improvements resulted from the individual effect of each TF in the cell behaviour (i.e., MYC-growth and XBP1s-productivity). An evaluation of the CHOCX_EPO_ cells under different environmental conditions (temperature and media glucose concentration) indicated that CHOCX_EPO_ cells increased cell productivity in high glucose concentration. This study showed the potential of combining TF-based cell engineering and process optimisation for increasing CHO cell productivity.

## Introduction

Recombinant proteins have become one of the most prominent therapeutic successes for the biopharmaceutical industry. An important part of this success has been due to advances in manufacturing technology, particularly the establishment of the Chinese hamster ovary (CHO) cell platforms that enable high production titres with adequate critical quality attributes (e.g., glycosylation)^1^. Whilst current CHO cell-based processes have surpassed the milestone of a multigram per litre (1–10 g/L)^2–4^, these production values are associated with a narrow number of molecules (mainly monoclonal antibodies [mAbs]). A significant number of complex biopharmaceuticals remain difficult to produce and achieve considerably low production titres. Several studies have associated poor product yields with a limited capacity of CHO cells to synthesise, process and secrete glycoproteins (what we call ‘productivity phenotype’)^5^. Enhancing CHO cell productivity (particularly for emerging formats) requires a greater understanding of cellular limitations and potential redesign of the molecular machinery involved in the synthesis and processing of non-standard-mAb molecular formats.

A powerful approach for engineering the CHO cell machinery and improving its productivity phenotype is the use of transcription factors (TFs)—regulatory molecules capable of gene activation/repression of multiple targets and alteration of cellular phenotypes^6^. In our previous studies, we have identified that the expression of two TFs (MYC and X-box binding protein 1, or XBP1) strongly correlated with product titres in CHO cell cultures^7,8^. MYC is a proto-oncogene that regulates many genes involved in cell proliferation, carbon metabolism and apoptosis, and its upregulation is a common feature in many different types of cancer cells^9,10^. Overexpression of MYC in CHO cells has led to increased viable cell densities and increased consumption rates for glucose and glutamine^11,12^, features that in other recombinant CHO cell lines have correlated with higher product titres. High expression of MYC leads to significant changes in cellular transcriptome and proteome, producing profiles relevant to the bioprocess effectiveness of CHO cells. For instance, CHO cells overexpressing MYC upregulated genes/proteins associated with glucose transporters, glycolytic enzymes and glutaminase^13–15^. High MYC expressing cells also showed an increase in protein synthesis rates through mechanisms that involve activation of mechanistic target of rapamycin (mTOR) signalling pathway, ribosome biogenesis and upregulation of eukaryotic initiation factors^13,16,17^. Although MYC overexpression in CHO cells has been shown to have positive consequences on cell proliferation and metabolism, its effects on commercially relevant recombinant protein production remain to be evaluated.

XBP1 is a key regulator of the endoplasmic reticulum (ER) protein processing and the unfolded protein response (UPR). In its active form (XBP1s,‘s’ reflecting spliced mRNA), it upregulates several ER-resident chaperones, enzymes involved in lipid metabolism and ERAD genes that increase the protein processing and secretory capacity of cells^18^. Upregulation of XBP1s results in expanded ER size and is critical for high-level antibody secretion rates in plasma cells^19^. In CHO cells, overexpression of XBP1s has been one of the most studied genetic modifications, and its effects have proven to be rather controversial. Previous studies have shown mixed results in the XBP1s effectiveness in increasing product titres and cell-specific productivity in CHO cells^5,6^. Currently, we lack understanding of the molecular mechanisms underlying the increased recombinant protein production in XBP1s overexpressing CHO cell lines. A generic proposed hypothesis envisages secretory bottlenecks (particularly at ER level) that are “loosened” by the multiple gene expression changes engaged by XBP1s action. This hypothesis would explain in particular increased production of recombinant molecules that are more challenging to produce (which expose the secretory bottlenecks more vigorously)^20–22^. In our previous study, we showed that XBP1s overexpression was effective when combined with an auxiliary transcription factor, B-lymphocyte-induced maturation protein 1 (BLIMP1). As BLIMP1 increased CHO cell protein synthesis, XBP1s overexpression solved the additional secretory pressure at the ER level^23^. These studies suggest that the effectiveness of XBP1s on recombinant protein productivity in CHO cells requires additional factors capable of increasing the cellular biosynthetic capacities. Here, we hypothesized that co-overexpression of MYC (as an auxiliary factor with the potential of increasing protein synthesis) and XBP1s will synergize to enhance recombinant protein production in CHO cells.

In this study, we investigated the effect of MYC and XBP1s co-overexpression on cell growth, recombinant protein production and cell metabolism in CHO cell line expressing a human erythropoietin (EPO). To address this objective, we generated an EPO producing CHO cell line (CHO_EPO_) using a targeted integrated (TI) approach, also known as recombinase-mediated cassette exchange (RMCE), to deliver the transgene into a predetermined site of the genome. Later, we engineered the CHO_EPO_ cell line to co-overexpress human MYC and XBP1s genes (CHOCX_EPO_). Single MYC or XBP1s overexpressing cell lines (CHOC_EPO_ and CHOX_EPO_) were also generated as additional controls. We characterised all the cell lines and compared them to the control (CHO_EPO_) in terms of culture performance, expression of recombinant genes and culture metabolism. To optimise the culture process, we evaluated the performance of the CHOCX_EPO_ and CHO_EPO_ cells under different conditions of temperature (33 and 37 °C) and initial medium glucose concentration (20 and 40 mM). Overall, we showed that a cell engineering strategy based on MYC and XBP1s was effective for increasing EPO production in CHO cells, particularly in cultures supplemented with high glucose.

## Results

### Generation of an EPO producing cell line

**Figure 1:**
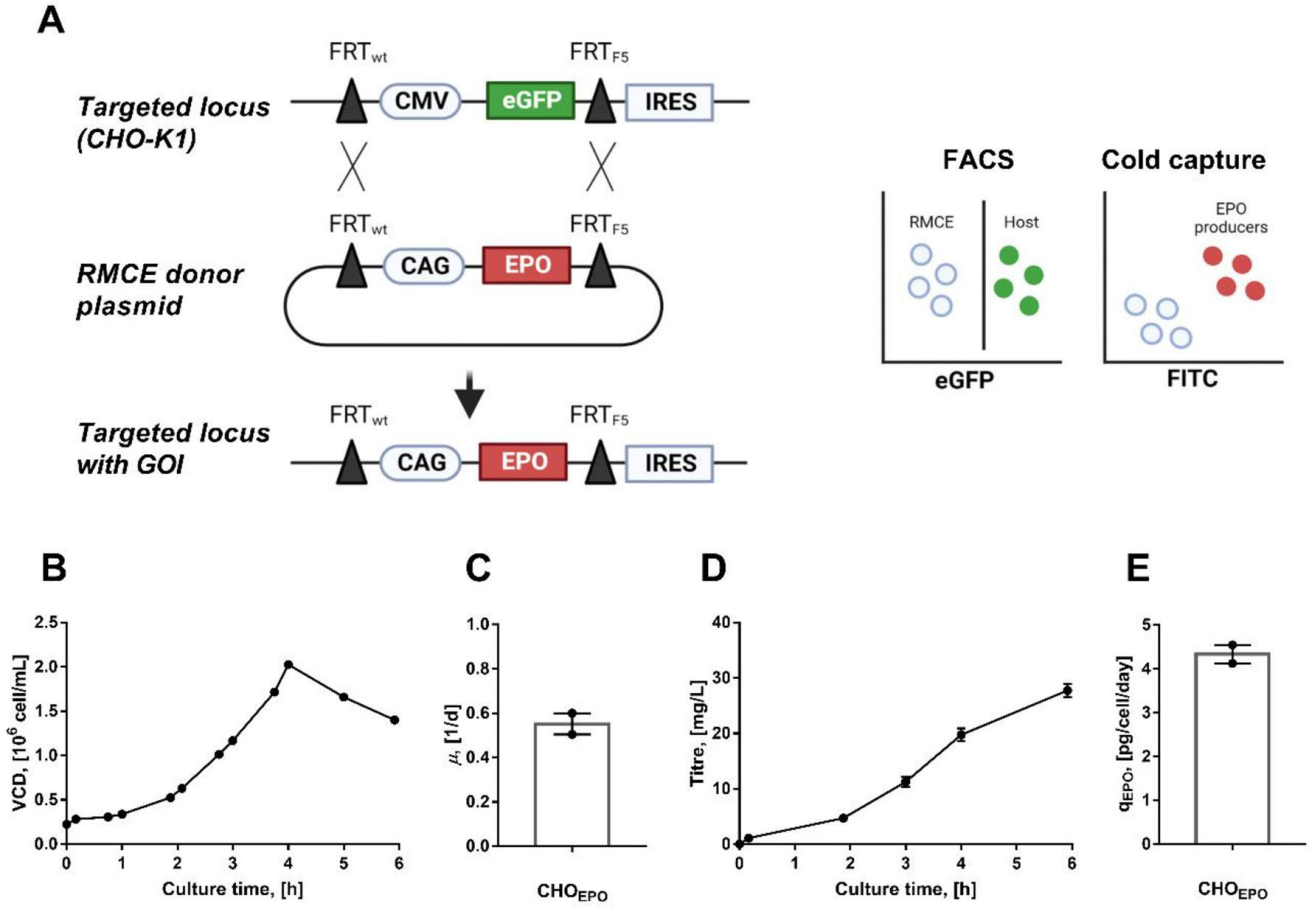
Generation of an EPO producing CHO cell line using recombinase-mediated cassette exchange (RMCE) system. (**A**) Overview of the RMCE-based cell line development. A CHO-K1 host cell line with an integrated, FRT and F5 flanked CMV-eGFP cassette^24^ was used as a master host. The master host was transfected with an RMCE donor plasmid (encoding for EPO) together with a plasmid encoding Flp recombinase for a site-specific recombination between FRT and F5 sites, respectively. In the resulting cell line the CAG-EPO cassette is integrated in the target locus. Dual FACS strategy was used for selecting targeted, eGFP-negative cells and FITC-positive cells for EPO antibody-based cold capture. (**B**) Viable cell density. (**C**) Cell-specific growth rate. (D) EPO titre profile. (E) Cell-specific productivity. Experimental values represent the mean of two biological replicates ± SEM.

Initially, we generated a recombinant EPO-producing cell line using a clonally derived CHO host cell line with a single copy of a Flp/FRT RMCE compatible tagging vector^24^. The original cassette (CMV-eGFP) in the master host cell line was exchanged by a CAG promoter-driven EPO cassette by a Flp-based targeting vector (Fig. 1A). Eight single-cell derived, eGFP negative clones were established by FACS and subjected to a sodium butyrate treatment to evaluate eGFP expression (potentially silenced through epigenetic mechanisms). A screening for eGFP signal showed different degrees of eGFP expression in several clones, except for clone 3 that presented both EPO production and no eGPF signal (Figure S1). Clone 3 was adapted to serum-free medium and suspension conditions. Characterisation of the clone 3 (or CHO_EPO_) showed a peak of cell density in day four and a cell-specific growth rate of 0.5 1/d (Fig. 1B,C). The CHO_EPO_ cell line presented maximum EPO production of about 30 mg/L in a six days batch culture, and a cell-specific productivity (q_EPO_) of 4.1 pg/cell/day (Fig. 1D,E). The production level of the CHOEPO cell line was comparable with previous reports of other CHO cell lines producing recombinant EPO (16–47 mg/L)^25–27^.

### Engineering CHO_EPO_ for overexpressing MYC and XBP1s

**Figure 2:**
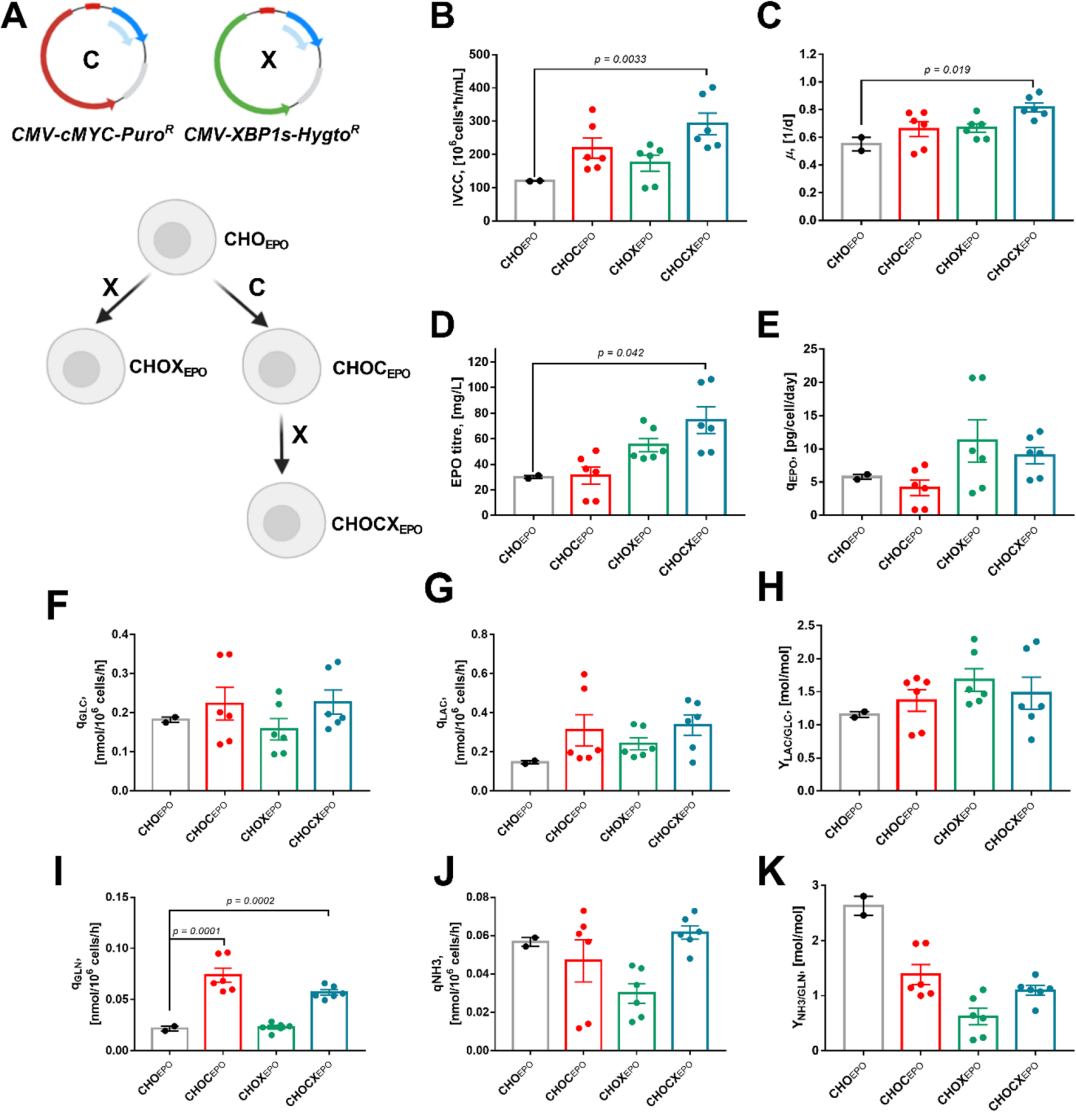
Development of a MYC and XBP1s overexpressing CHO cell line. (*black*) CHOEPO cell line; (*red*) CHOC_EPO_ cell line; (*green*) CHOX_EPO_ cell line; (*blue*) CHOCX_EPO_ cell line. (**A**) Overview of the cell engineering strategy to overexpress MYC and XBP1s in the CHO_EPO_ cell line. The CHOXC_EPO_ cell line was generated by transfecting cells with the CMV-MYC-Puro^R^ and the CMV-XBP1s-Hygro^R^ plasmid, and isolating cells resistant to puromycin and hygromycin in the culture medium. Additionally, the CHOC_EPO_ and CHOX_EPO_ cell lines were generated as control cell lines. (**B**) Integral of viable cell concentration (IVCC). (**C**) cell-specific growth rate (µ). (**D**) EPO titre. (**E**) cell-specific EPO productivity (q_EPO_). (**F**) Glucose consumption rate (q_GLC_). (**G**) Lactate production rate (q_LAC_). (**H**) Ratio of lactate produced to glucose consumed (Y_LAC/GLC_). (**I**) Glutamine consumption rate (q_GLN_). (**J**) Ammonia production rate (q_NH3_). (**K**) Ratio of ammonia produced to glutamine consumed (Y_NH3/GLN_). Each dot corresponds to the duplicates (n = 2) of the three isolated cell clones. *p* values (One-way ANOVA) below to 0.05 indicate the statistical significance of parameter variation.

We engineered the CHO_EPO_ cell line to co-overexpress MYC and the functional form of XBP1 (or XBP1s) as a strategy for enhancing EPO production. To achieve this goal, we transfected the CHO_EPO_ cell line with two expression vectors containing MYC and XBP1s human sequences and selected for the growth of a heterogeneous population (pools) resistant to puromycin and hygromycin (CHOCX_EPO_) (Fig. 2A). Single MYC or XBP1s overexpressing CHO_EPO_ cell lines (CHOC_EPO_ and CHOX_EPO_) were also generated as additional controls. We isolated three single-cell clones from the mixed population and characterised their cell growth, EPO production and culture metabolites during standard batch cultures (Fig. 2B–E). Overall, we found that the co-expression of recombinant MYC and XBP1s genes significantly improved the CHO_EPO_ cell performance and reshaped metabolism in culture.

We observed that the CHOCX_EPO_-derived clones reached significantly higher cell densities (defined as the integral of viable cell concentration [IVCC]) and faster cell-specific growth rates that the control CHO_EPO_ cell line (Fig. 2B,C). However, we did not find statistical differences between the CHOCX_EPO_ cells and the single MYC and XBP1s overexpressing cells. The increased cell growth in the CHOCX_EPO_ cells was associated with the MYC overexpression, an improvement that has previously observed in other CHO cell lines^11^. In terms of EPO production, we found that CHOCX_EPO_ cell lines had significantly higher EPO titres compared to the other cell lines, with an increase of 2.5-fold on average EPO production compared to the original CHO_EPO_ (Fig. 2D). We also observed a slight increased cell-specific productivity in the XBP1s overexpressing cell lines (both CHOX_EPO_ and CHOCX_EPO_), although these differences were not statistically relevant (Fig. 2E). This discrepancy between the EPO titres and q_P_ in the CHOCX_EPO_ cells might be explained by the increased IVCCs due to MYC overexpression.

We also evaluated culture metabolites (glucose, lactate, glutamine and ammonia) and calculated their cell-specific rates of consumption/production to compare the impact of MYC and XBP1s overexpression on cell metabolism (Fig. 2F–K). We found that MYC overexpression had a great influence on the metabolic behaviour of our cells. The CHOCX_EPO_ cells increased their lactate production rates (q_LAC_), despite of maintaining similar glucose consumption rates (q_GLC_) compared to the control. Additionally, we observed that the CHOCX_EPO_ cells increased glutamine consumption rates (q_GLN_), without significantly varying ammonia production rates (q_NH3_) compared to the control. The increased consumption of glutamine and production of lactate were associated with MYC overexpression, as the CHOC_EPO_ cells also presented similar increase in glutamine and lactate metabolism. These data suggested that most of the overproduction of lactate in MYC overexpressing cells resulted from the high consumption of glutamine. The glutamine-driven lactate production is a phenomenon previously reported in CHO cells, in which a significant part of the glutamine is metabolised in the TCA cycle (oxidised to malate) and then converted into pyruvate in the cytosol^28,29^.

### Gene expression of recombinant EPO, MYC and XBP1s and its relationship to product titres and cell proliferation

**Figure 3:**
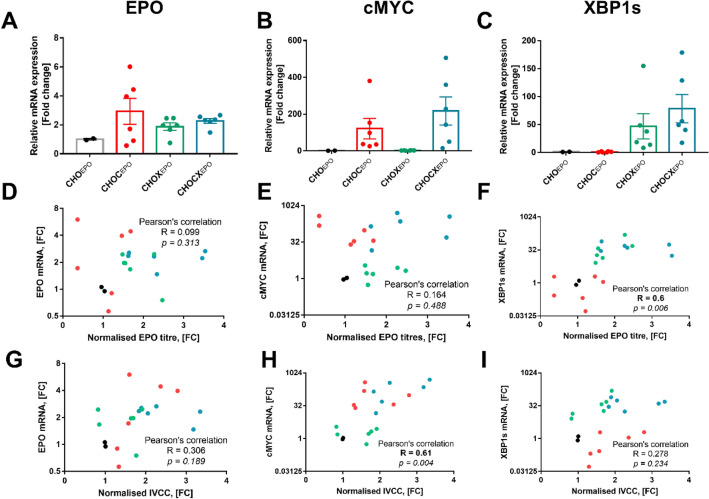
Relative mRNA expression of recombinant EPO, MYC and XBP1 and their relationship with recombinant EPO production. (*black*) CHOEPO cell line; (*red*) CHOC_EPO_ cell line; (*green*) CHOX_EPO_ cell line; (*blue*) CHOCX_EPO_ cell line. (**A**,**B**) and C) mRNA expression of EPO, MYC and XBP1, respectively, normalised using GAPDH as internal standard. Samples for mRNA expression analysis correspond to day 3. (**D**–**F**) Comparison between normalised EPO titres (*x*-axis) and mRNA levels of EPO, MYC and XBP1s (*y*-axis), respectively. (**G**–**I**) Comparison between normalised IVCC titres (*x*-axis) and mRNA levels of EPO, MYC and XBP1s (*y*-axis), respectively. Each dot corresponds to a replicate derived from CHOEPO cells as well as from three independent single-cell derived CHOC_EPO_ , CHOX_EPO_ and CHOCX_EPO_ cell lines.

Next, we evaluated whether improvements in EPO production and cell growth in MYC and XBP1s transfected cells were associated with variations in recombinant gene expression (Fig. 3). We confirmed strongly increased mRNA expression of MYC and XBP1s in the respectively engineered cell lines (Fig. 3B,C). However, we only found minor, statistically non-significant differences in EPO mRNA expression, indicating that the TF did not increase CAG promoter driven EPO transcription (Fig. 3A).

To evaluate the influence of the targets (i.e., EPO, MYC and XBP1s) on EPO production and cell growth, we performed statistical Pearson’s correlations between the normalised EPO titres or IVCC and the mRNA expression of the recombinant EPO, MYC and XBP1s genes (Fig. 3D–I). Whilst we found no relationship between the mRNA expression of the recombinant EPO and/or MYC genes and EPO productivity (Fig. 3D,E), the XBP1s mRNA expression presented a slight correlation with the EPO titres (Pearson’s correlation, R = 0.6, *p* = 0.006) (Fig. 3F). Moreover, we only observed a slight correlation between the MYC mRNA levels and the IVCC (Pearson’s correlation, R = 0.61, *p* = 0.004) (Fig. 3H), while the mRNA expression of EPO and XBP1s did not present any relationship with cell growth variations of the cell lines (Fig. 3G–I). These results indicated that MYC and XBP1s overexpression had a positive influence on cell proliferation (Figs. 2B,C, 3H) and EPO secretion in our engineered CHO cell lines, respectively (Figs. 2D,E, and 3F). It is also interesting to note that XBP1s overexpression was more effective for increasing EPO production when co-expressed with MYC (Figs. 2D and 3F).

### Process optimisation for the CHOCX_EPO_ cell line: glucose and temperature

**Figure 4:**
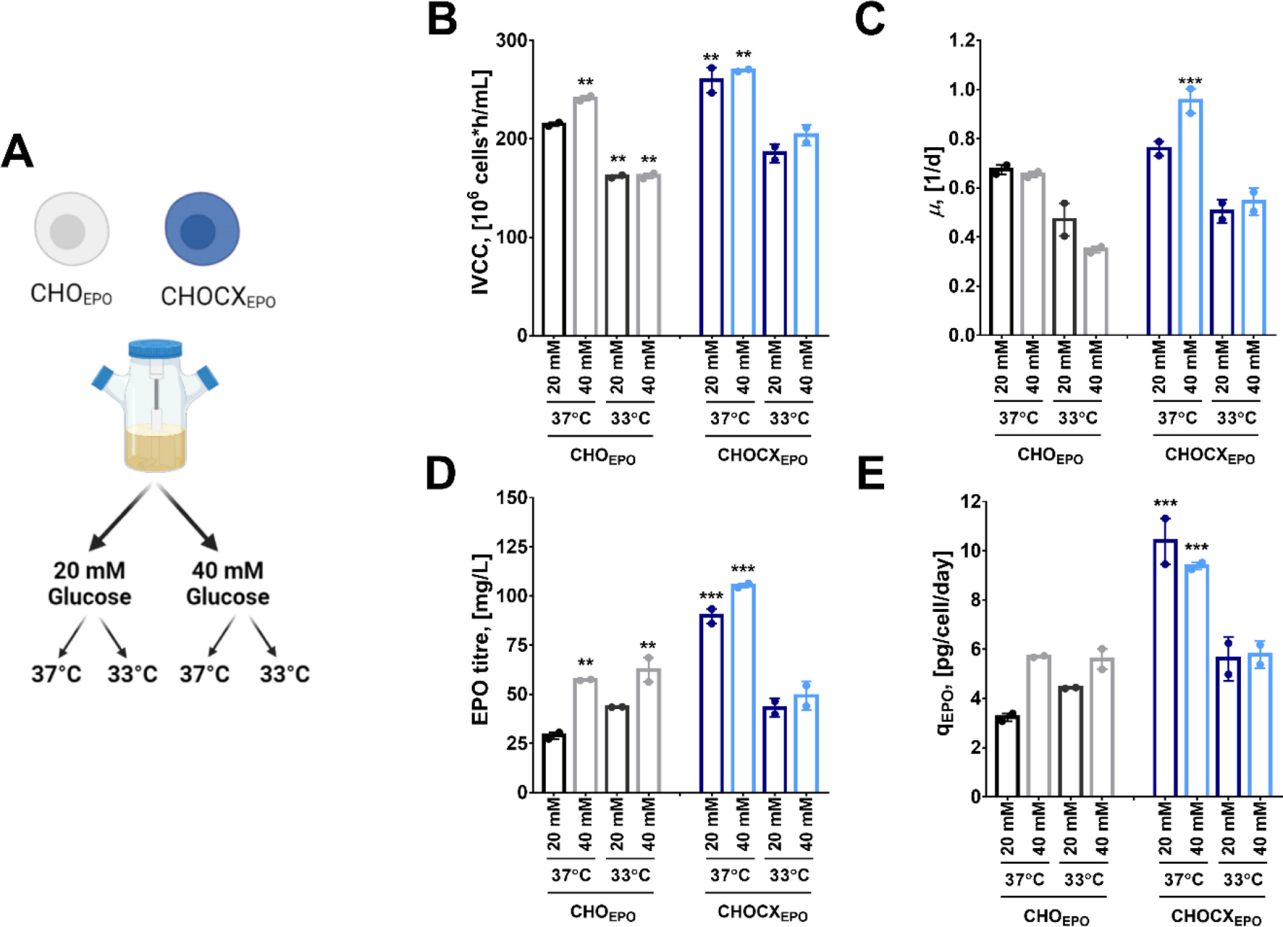
Comparison of glucose and temperature effect on the culture performance of CHO_EPO_ and CHOCX_EPO_ cell lines. (*black*) CHO_EPO_ in 20 mM glucose, (*grey*) CHO_EPO_ in 40 mM glucose, (*dark blue*) CHOCX_EPO_ in 20 mM glucose; (*light blue*) CHOCX_EPO_ in 40 mM glucose. (**A**) Overview of the culture conditions evaluated for both cell lines. (**B**) Integral of viable cell concentration. (**C**) Cell-specific growth rate. (**D**) EPO titre. (**E**) Cell-specific productivity. Experimental values represent the mean of two biological replicates ± SEM. (*), (**) and (***) indicated *p* < 0.05, 0.001, 0.0001 (*Two-way* ANOVA). Culture of CHO_EPO_ at 37 °C and 20 mM glucose was the control condition.

In the previous sections, we observed that the CHOCX_EPO_ cells presented significantly better performance (i.e., simultaneously higher cell growth and productivity) than the other cell lines. However, we characterised the engineered cell lines using cultures at 37 °C and supplemented with 20 mM glucose, two parameters that can be optimized for increased performance of CHO cells in cultures^30^. To address this point, we evaluated the growth, EPO production and culture metabolites of the best producer CHOCX_EPO_ clone and the control CHO_EPO_ cells under different conditions of culture temperature (37 and 33 °C) and initial glucose concentration (20 and 40 mM) using spinner flasks. Overall, we found that decreasing culture temperature and increasing initial glucose concentration in media had a significant impact on culture performance and metabolic behaviour (Fig. 4 and Table 1).

**Table 1:** Metabolic parameters of the CHO_EPO_ and CHOCX_EPO_ at different culture temperatures (37 and 33 °C) and initial glucose concentrations (20 and 40 mM).

	Unit	CHO_EPO_				CHOCX_EPO_			
		37 °C		33 °C		37 °C		33 °C	
		20 mM	40 mM	20 mM	40 mM	20 mM	40 mM	20 mM	40 mM
q_GLC_	pmol/cell/day	4.5 ± 0.07	2.3 ± 0.06*	1.9 ± 0.02*	1.5 ± 0.01*	3.8 ± 0.30	8.5 ± 0.33*	3.8 ± 0.24	2.9 ± 0.17*
q_LAC_	pmol/cell/day	3.7 ± 0.27	5.6 ± 0.08*	1.1 ± 0.06*	1.8 ± 0.02*	4.8 ± 0.20	9.9 ± 0.40*	3.1 ± 0.68	1.9 ± 0.25*
Y_LAC/GLC_	mol/mol	0.8	2.4	0.6	1.2	1.3	1.2	0.8	0.6
q_GLN_	pmol/cell/day	0.4 ± 0.01	1.1 ± 0.02*	0.6 ± 0.03*	0.7 ± 0.03*	1.4 ± 0.11*	1.5 ± 0.03*	0.8 ± 0.02*	0.7 ± 0.05*
q_NH3_	pmol/cell/day	1.2 ± 0.00	1.2 ± 0.06	0.8 ± 0.06*	1.1 ± 0.02*	1.8 ± 0.16	0.6 ± 0.01*	0.3 ± 0.07*	0.4 ± 0.06*
Y_NH3/GLN_	mol/mol	2.9	1.1	1.3	1.6	1.3	0.4	0.4	0.6

(*) indicates *p*-values (*t*-test) below 0.05 (Culture of CHO_EPO_ at 37 °C and 20 mM glucose was the control condition).

We found that low temperature decreased cell densities and cell-specific growth rates, both in the CHO_EPO_ and CHOCX_EPO_ cells, as expected (Fig. 4B,C). In contrast, decreasing culture temperature had differential cell line-specific effects on EPO production (Fig. 4D,E). The CHO_EPO_ cells presented increased EPO titres and q_P_ in cultures at 33 °C, a response that is commonly observed in CHO cell cultures^7,8^. However, the CHOCX_EPO_ cells decreased EPO production and cell productivities, regardless of the concentration of glucose.. In addition to changes in growth and productivity, low temperature led to a significant decrease in glucose and glutamine consumption as well as lactate production in both the CHO_EPO_ and CHOCX_EPO_ cells (Table 1). This decrease in carbon metabolism was consistent with other studies on CHO cell cultures at low temperature^7,31^.

High glucose concentration increased cell densities, regardless of the specific cell lines, although it only increased cell-specific growth rates in the CHOCX_EPO_ cells (Fig. 4B,C). It is interesting to note that high glucose concentrations had no significant effect on cell growth in cultures at 33 °C, a phenomenon that might be associated with the growth-inhibiting effects of low temperature. We also found that high glucose concentration increased EPO titres (Fig. 4D), leading to the most productive condition (40 mM and 37 °C) in both the CHO_EPO_ and CHOCX_EPO_ cells. In contrast to low-temperature cultures, the CHO_EPO_ and CHOCX_EPO_ cells had higher consumption of glucose and glutamine, and production of lactate in cultures with 40 mM compared to those with 20 mM. The CHOCX_EPO_ cells in cultures at 37 °C and 40 mM glucose was the condition that presented the highest consumption rates of glucose and glutamine. These data also agree with previous studies showing the relevance of glucose and glutamine in recombinant protein production in CHO cells^30,32^. Whilst this response of the CHOCX_EPO_ cells seemed to be a consequence of overexpressing MYC, a transcription factor involved in the activation of carbon metabolism, it revealed the importance of glucose and glutamine metabolism on the overall performance of CHO cells.

**Figure 5:**
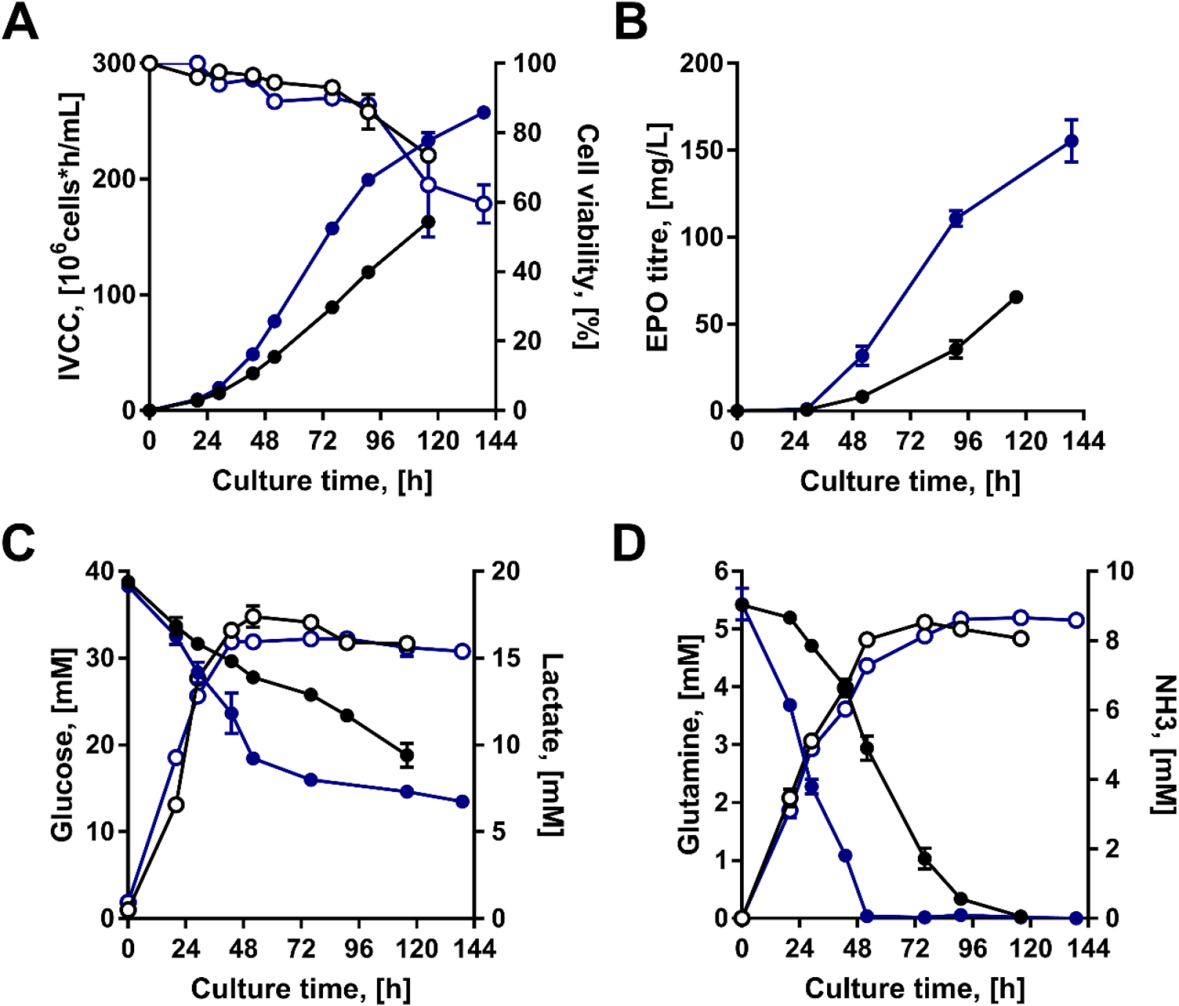
Comparison of the CHO_EPO_ and CHOCX_EPO_ cell lines in controlled bioreactors with 40 mM glucose and at 37C. (*black*) CHO_EPO_, (*blue*) CHOCX_EPO_. (**A**) Integral of viable cell concentration (IVCC, filled circle) and cell viability percentage (light circle). (**B**) EPO titres. (**C**) Glucose (filled circle) and lactate profiles (light circle). (**D**) Glutamine (filled circle) and ammonia profiles (light circle). Experimental values represent the mean of two biological replicates ± SEM.

Lastly, we further investigated the performance of the CHOCX_EPO_ and the control CHO_EPO_ cells in controlled bioreactors, a commonly used scale-down model for larger-scale manufacturing settings. We focussed on the most productive condition determined in our previous experiments (40 mM and 37 °C). As expected, we observed that the CHOCX_EPO_ and CHO_EPO_ cells had a better performance in a controlled bioreactor than in spinner flasks (Fig. 5). The CHOCX_EPO_ cells had a higher peak of cell density (1.7-fold increase) and EPO production (2.4-fold increase) than our control. This increase in cell density and EPO production coincided with the increased consumption of glucose and glutamine in our CHOCX_EPO_ cells. It is worth noticing that rapid glutamine consumption did not result in higher production of lactate and ammonia in our CHOCX_EPO_ cells, as it occurred in our spinner flask cultures. However, apart from the lactate profile, the results in bioreactors were consistent with our initial results from spinner flasks. Together, this demonstrates that our cell engineering strategy based on MYC and XBP1s effectively improved the overall performance of CHO cells for manufacturing EPO.

## Discussion

CHO cells are robust and well-established production platforms for the production of protein therapeutics. However, their limited productivity phenotype is still a challenge to overcome. There has been an increasing interest in engineering the CHO cell machinery to make these cell factories more robust protein factories. Here, we addressed the necessity of improving CHO cell productivity phenotypes by co-expressing two TFs involved in cell proliferation/metabolism (MYC) and protein processing/secretion (XBP1s). We demonstrated the effectiveness of overexpressing MYC and XBP1s for enhancing overall culture performance of CHO cells by a simultaneous increase in viable cell densities and product yields (Fig. 2). These changes resulted from the individual effects of each TF, where MYC increased cell growth, while overexpression of XBP1s improved EPO productivity (Fig. 3F,H).

A concomitant enhancement of cell growth and recombinant protein production is a desired goal for CHO cell engineering, but such an outcome has been very difficult to achieve through genetic manipulations. A limited number of studies have achieved this goal, in which mitochondrial/energy metabolism seemed to play a central role. For instance, CHO cells overexpressing key regulatory elements of carbon and energy metabolism (e.g., mTOR or Forkhead box protein A1 [Foxa1]) have achieved large improvement in viable cell densities and recombinant protein production^33,34^. Using an enrichment approach based on a high mitochondrial membrane potential (MMP), a new CHO cell host (derived from a CHOK1 host) with increased cell densities, cell viability and productivity of different ‘difficult-to-express’ proteins was developed^35,36^. Supplementing culture medium with copper have also led to simultaneous increase in growth and productivity in CHO cells^37^, through mechanisms that that have been proposed to involve enhanced oxidative metabolism^38,39^. In agreement with these studies, high expressing MYC or XBP1s cells have also presented an increased mitochondrial metabolism^23,40^, a response that might have benefited the performance of our CHOCX_EPO_ cells (Fig. 3). Although potential changes in energy metabolism need further investigations, we showed that combining two TFs (associated with different cellular processes) were effective for improvement of the productivity phenotype of CHO cells. Future work will need to examine the possibility to multiplexed TF-based cell engineering that can reshape CHO cells (or other mammalian cell host) towards very-high level protein-producing factories.

In the diverse portfolio of cell engineering strategies, the overexpression of XBP1 (particularly its spliced form) has been one that has captured the greatest attention because of its key regulatory role of the secretory pathways. Several studies have engineered CHO cells to overexpress XBP1s, leading to different results in protein secretion (null to fourfold improvements)^22,23,41,42^. Previous studies have suggested that the effectiveness of XBP1 overexpression depended on the ER stress levels imposed by the r-protein synthesis^5,21,43^. Here, we found that co-expression of XBP1s with MYC significantly increased EPO titres in CHO cells (Fig. 2). These improvements resulted from the combinatorial effect of these two TFs. While MYC increased the number of cells in the bioreactor (Fig. 3H), XBP1 expression correlated with the amounts of secreted EPO (Fig. 3F). We hypothesised that MYC overexpression presented a positive auxiliary factor for XBP1s for enhancing the EPO production in CHO cells. This hypothesis agrees with other studies showing the effectiveness of XBP1 overexpression when combined with other transcription factors^23^. However, it remains to be further investigated whether the combination of XBP1s with a large number of TFs can promote larger improvements in CHO cell performance.

We also found that our engineered CHO cells had an increased performance under high glucose concentration medium in standard culture conditions (but not under low temperature conditions) (Fig. 4). The cellular responses to a varied glucose environment and temperature have been shown to be highly dependent of the specific cell line and culture conditions. In the case of glucose availability, CHO cells in continuous culture presented higher cell densities and recombinant protein production under high glucose conditions^30^. However, glucose concentration in medium (when it is not depleted) had minimal effects on cell performance in fed-batch cultures^44,45^, particularly because cells rely on other metabolites (e.g., amino acids) in later stages of the culture. Our cultures were supplemented with glucose from the start, which could also make the difference from fed-batch cultures that include glucose feeding over time. In the case of culture temperature, our experience has shown us that recombinant CHO cell lines have different responses to a decrease in culture temperature, a phenomenon that related to its global impact on CHO cell transcriptome and proteome^46–48^. In particular, our data suggest that the overexpression of MYC and XBP1s had a large influence on the response of the CHOCX_EPO_ cells to varied glucose and temperature environment. For instance, high MYC expressing cells have been shown to have increased glycolytic fluxes and glutamine breakdown^40,49^, a metabolic profile that often correlated with high proliferation and lactate production^29^. It seems possible that MYC overexpression may make CHO cells more responsive to a high glucose environment, as we observed in our CHOCX_EPO_ and CHOC_EPO_ cells (Fig. 2). In contrast, the poor performance of our CHOCX_EPO_ cells in low temperature cultures was certainly unexpected, particularly as temperature change was effective in increasing productivities in the CHO_EPO_ cells. Low temperature often slows down carbon metabolism, with a decrease glucose/glutamine consumption as a common features in CHO cells^7,50,51^. This response to low temperature might have conflicted with other effects generated by overexpression of MYC, which presented higher consumption of glucose and glutamine (Fig. 2). Previous studies have also shown that low temperature upregulated XBP1s as well as their specific targets^7,8^. Changes in XBP1s expression at low temperature in our CHOCX_EPO_ cells might have resulted in disadvantageous changes (e.g., induction of apoptosis, decrease in protein synthesis) that impaired their culture performance. We need to further investigate the effect of low temperature in both single MYC and/or XBP1s overexpressing cells to determine whether the decreased performance linked to the expression of these two TFs. However, the fact remains that a thorough process optimisation is required to find the best conditions for maximising the recombinant protein production in genetically engineered cells and the optimal conditions may vary from any expectations built around standard cell platforms.

This study has provided novel insights into the effectiveness of combining a cell and process engineering approached to enhance recombinant EPO production in CHO cells. However, the present results must be interpreted with caution, and potential limitations need to be considered. An initial limitation considers the relevance of the clonal effect during our cell line development (CLD) and engineering processes. Given the nature of single cell cloning, cells undergo changes (at genetic, epigenetic and transcriptomic levels) that might have affected their capacity to growth and secrete the r-proteins^52^. Although the limited number of clones evaluated for each cell line (3 for each engineering approach) might not provide a full separation of the consequences of overexpressing these two TFs’ from the clonal effect, our data showed clear correlations between the expression levels of MYC and XBP1s with cell growth and EPO titres, respectively, in CHO cells. A further investigation is required to evaluate whether this dual cell engineering can consistently enhance the performance of other complex r-proteins and in a large number of clones.

Another important questions remain to be answered of the extent to which TF-based cell engineering led to the up-/downregulation of the specific TF’s targets. The effects of TFs on cellular transcriptome (and phenotype) have proven to be dependent on their expression levels^53^, thus raising the question about the ‘optimal’ TF expression dosage. Different studies have attempted to answer this question using transient expression experiments. However, CHO cells present low efficiency for transient transfection that often leads to discrepancies between transiently and stably expressing cell lines^22^. Addressing this question requires a deeper understanding of the effects of MYC/XBP1s (or other engineering strategies) on the regulation of transcriptome, proteome and metabolome of CHO cells. A more profound characterisation of the CHOCX_EPO_ cells at a molecular level will offer relevant insights for the development of CHO cell lines capable of decoupling cell growth and productivity in the context of bioprocessing. Lastly, we are unaware of the consequences of a simultaneous increase in cell growth and proliferation on the recombinant protein quality of our engineered cell lines. From an industrial perspective, an increase in cell productivity must be accompanied by the best product quality possible. Further investigation of the impact of MYC and XBP1s on the recombinant protein glycosylation and other critical quality attributes is critical for translating this technology to manufacturing settings.

In summary, our findings reveal that TF-based cell engineering has the potential for reshape CHO cell phenotypes towards an increased productive capacity. The co-overexpression of MYC and XBP1s led to a simultaneous increase in viable cell densities and recombinant protein production, an improved productivity phenotype that linked to the expression of these two TFs. To the best of our knowledge, this is the first study evaluating the combined expression of MYC and XBP1s as a strategy for enhancing CHO cell performance for manufacturing recombinant proteins. From a general perspective, these findings offer an attractive approach to maximise both cell growth and productivity in CHO cells simultaneously, an approach that could be used for improving product yields of recombinant CHO cells during industrial cell line development. The CHOCX_EPO_ cell line can also serve as a model to engineer cells suited as hosts for high-density culture in intensified upstream processes.

## Methods

### Plasmid design and construction

Standard molecular cloning techniques were used to construct the plasmids for this study. A Flp/FRT targeting vector containing a recombinant human erythropoietin (hEPO) was constructed by inserting the EPO sequence from pLenti4/TO-hEPO plasmid (Addgene #50437^54^) into the *AgeI* and *NotI* sites of a CAG-MCS plasmid (referred as CAG-EPO), thereby controlling EPO expression by the CAG promoter (Fig. 1A). The hEPO expression cassette was flanked by a wild-type FRT site and F5 spacer mutant FRT site. The plasmid expressing the spliced form of XBP1 (or XBP1s) was constructed by sub-cloning hygromycin resistant gene into the *SmaI* and *XbaI* sites of the pCMV5-Flag-XBP1s plasmid (Addgene #63680^55^) (referred as CMV-XBP1s-Hygro^R^). The plasmid expressing human MYC was obtained from Addgene (#46970^56^) (referred to as CMV-MYC-Puro^R^). The XBP1s and MYC coding sequence are under the control of a human CMV promoter, with hygromycin and puromycin as the selection marker under the control of an EF1α promoter, respectively.

### Cell line development and engineering

A CHO-K1 cell line with an integrated landing pad (pTAG-CMV-eGFP) was used as a host in this study^24^. All cells were transfected using Lipofectamine 2000 as the manufacturer’s protocol. An EPO producing cell line (referred as CHO_EPO_) was generated using the Flp-based RMCE method as previously described^57^. Briefly, 1 × 10^6^ CHOK1 cells were co-transfected with 3 µg of the RMCE donor plasmid (CAG-EPO) and 1 µg the FLPe expression vector. After a 5 h incubation, cells were centrifuged and culture media was replaced with fresh medium. Cells were grown for 10 days under standard culture conditions (37 °C, 5% CO_2_, 95% humidity) and sorted using a BD FACSAria™ III Sorter (BD Biosciences). A dual-target FACS sorting strategy was used to sort negative eGFP cells (eGFP was originally present in the targeted locus) and positive EPO-producing cells using a cold-capture approach using EPO-FITC conjugated antibody (sc-80995, Santa Cruz Biotechnology) as previously described^58^. From the EPO-producing cell pool, eight single-cell clones were isolated. Sodium butyrate treatment was used to evaluate potential eGFP signals that could have been silenced in the eight isolated clones. The clone with no eGFP signal, and EPO gene integration (confirmed through PCR) was used for this study (referred to as CHO_EPO_) (Figure S1 and S1).

The CHO_EPO_ cells were transfected with 3 µg of the CMV-MYC-Puro^R^ and/or 3 µg of the CMV-XBP1s-Hygro^R^ plasmids to generate MYC and XBP1s overexpressing cell lines (referred to as CHOC_EPO_, CHOX_EPO_ and CHOCX_EPO_). After 72 h incubation in growth medium, cells were exposed to the corresponding selection medium (10 μg/mL Puromycin for MYC and/or 250 μg/ mL Hygromycin B for XBP1s). About three weeks after seeding, heterogeneous cell pools overexpressing MYC and/or XBP1s were recovered, and samples for the assessment of recombinant EPO, MYC and XBP1s gene integrations were taken. The CHOC_EPO_, CHOX_EPO_ and CHOCX_EPO_ cells were subjected to single-cell cloning, and three clones from each population were randomly selected. Frozen cell stocks were made for further analysis when the CHO_EPO_, CHOC_EPO_, CHOX_EPO_ and CHOCX_EPO_ cells were adapted to a suspension/serum-free medium ("Gene expression of recombinant EPO, MYC and XBP1s and its relationship to product titres and cell proliferation" section) and presented 100% viability.

### Cell culture and media

The CHOK1 host cells were grown in 1:1 ratio of DMEM and Ham’s-F12 media (Invitrogen), supplemented with 5% fetal bovine serum (FBS), 2 mM glutamine (Sigma), 0.5 mM sodium pyruvate (Sigma) and Penicillin–Streptomycin (10 U/mL, Gibco). All CHOK1-derived cell lines (i.e., CHO_EPO_, CHOC_EPO_, CHOX_EPO_ and CHOCX_EPO_) were gradually adapted to serum-free HyClone CDM4CHO cell culture medium (Cytiva) and suspension conditions.

Cells were routinely subcultured every 48 h in batch systems using T-75 flasks and scaled up in spinner flasks (Techne) by reseeding at 2 × 10^5^ cells/mL in fresh media. To evaluate the performance of the CHO_EPO_, CHOC_EPO_, CHOX_EPO_ and CHOCX_EPO_ cell lines, all cultures were performed in batch systems using spinner flasks. To evaluate the effect of glucose concentration conditions, the control CHO_EPO_ cells and the most productive CHOCX_EPO_ clone (“Gene expression of recombinant EPO, MYC and XBP1s and its relationship to product titres and cell proliferation” section) were cultured in batch systems using HyClone CDM4CHO cell culture medium (Cytiva) supplemented with 20 (Low) or 40 (High) mM glucose. To evaluate the effect of low temperature, the CHO_EPO_ and CHOCX_EPO_ cells were grown at 33 °C for three passages before undertaking batch culture as previously reported^30^. All cell cultures were performed in duplicate in 125 mL spinner flasks with a working volume of 60 mL growth medium, incubated in a Forma™ Series II 3110 Water-Jacketed CO_2_ Incubator (Thermo Fisher Scientific) with 96% humidity and 5% CO_2_ enriched atmosphere. Sampling was carried out every 24 h until viability decreased to 70%.

### Bioreactor operation

The performance of CHO_EPO_ and CHOCX_EPO_ cell lines was evaluated in batch cultivation using controlled Applikon MiniBio 500 bioreactors (Applikon Biotechnology). Bioreactors were seeded at 2 × 10^5^ cells/mL with an initial cell culture volume of 200 mL HyClone CDM4CHO cell culture medium, supplemented with 40 mM glucose and 6 mM glutamine (the best condition evaluated in “Process optimisation for the CHOCX_EPO_ cell line: glucose and temperature” section). The cultures were mixed using a vortex marine impeller rotating at 120 rpm and supplied with a constant airflow rate of 0.4 mL/min. The bioreactor operation was controlled using a *my*-control unit (Applikon Biotechnology), maintaining culture temperature at 37 °C with a heating blanket and pH 7.0 with CO_2_ supply and 100 mM NaHCO_3_Na_2_CO_3_ alkalli solution. Dissolved oxygen tension (DOT) was set to a minimum of 30%. The process parameters were continuously monitored using BioExpert software (v 1.1X, Applikon Biotechnologies). Samples were collected daily for cell counting, EPO measurements and metabolite analysis. Two bioreactor runs were conducted for each condition.

### Analytical methods

Cell concentration and viability were determined by a haemocytometer (Neubauer, Germany) using the method of trypan blue exclusion (Sigma). Glucose, lactate, glutamine, and glutamate concentrations were measured using an YSI 2700 Biochemistry Analyser (Yellow Springs Inc.). Glutamate concentration was measured to subtract the glutamate contribution in culture medium during the measurement of glutamine concentrations. Ammonia concentration was measured using Biosystems Analyser Y15 (BioSystems) and an Ammonia AX5 kit (#12532, BioSystems S.A.). EPO concentration in the medium was determined by a commercially available ELISA assay (ELH-EPO-1, RayBiotech), according to the manufacturer's protocol.

### Gene expression analysis by PCR and RT-qPCR

For RNA extraction, 5 × 10^6^ viable cells were harvested by centrifugation (500 g, 10 min). The cell pellets were lysed in 1 mL Trizol™ Reagent (Invitrogen) and processed according to the manufacturer’s instructions. The RNA concentration and purity were determined using a BioSpec-nano Micro-volume UV–Vis Spectrophotometer (Shimadzu). RNA samples with an A260/A280 ratio between 1.8 and 2.0 and an A260/A230 ratio > 2.0 were regarded as appropriate for analysis. Genomic DNA was removed through treatment with DNase I (NEB) and the resulting RNA was converted to complementary DNA (cDNA) using AffinityScript cDNA Synthesis Kit (Agilent) as per the manufacturer’s instructions. Real-time quantitative PCR (RT-qPCR) was carried out in an Agilent AriaMx Real-time PCR System (Agilent) using a Brilliant II SYBR^®^ Green QRT-PCR AffinityScript Master Mix (Agilent), according to the manufacturer’s instructions. All samples were run in duplicate. The thermal cycling parameters used consisted of 3 s at 95 °C as initial denaturation, followed by 40 cycles of 5 s at 95 °C and 20 s at 55 °C. A final extension at 72 °C was carried out, followed by a melting curve to confirm primer specificity. Data were analyzed using the 2^−ΔΔCT^ method and normalized using *β*-*actin* as a standard. The mRNA was quantified by using the following primers (*β-actin*: forward 5′-GCAAGCATCCCCCAAAGTTC-3′, reverse 5′-CTTTTGGGAGGGTGAGGGAC-3′; *epo*: forward 5′-TCATCTGTGACAGCCGAGTC-3′, reverse 5′-TCCATCCTCTTCCAGGCATA-3′; *myc*: forward 5′-TGCTGTCGTTGAGAGGGTAG-3′, reverse 5′-TCCAGGACTGTATGTGGAGC-3′; *xbp1s*: forward 5′-AGCTCAGACTGCCAGAGATC-3′, reverse 5′-TCACTTCATTCCCCTTGGCT-3′).

### Calculation of specific rates and statistical analysis

Cell-specific growth rate (μ) EPO productivity (q_EPO_) and metabolite consumption/production rates (q_GLC_, q_GLN_, q_LAC_ and q_NH3_) were calculated as described previously^2^. The ratio of lactate produced to glucose consumed (Y_LAC/GLC_) and the ratio of ammonia produced to glutamine consumed (Y_NH3/GLN_) were calculated using the cell-specific rates of each metabolite. All statistical analyses were performed in the R software (version 3.1). Statistical analysis of the physiological parameters was evaluated by Two-way ANOVA (using culture temperature and glucose as factors) followed by multiple comparison tests (Tukey HSD test) with normally distributed data. Variance homogeneity and normal distribution of residuals were assessed with the Shapiro–Wilk test to validate the ANOVA's assumptions. Significant differences in mRNA expression levels between the cell lines were evaluated by a t-test. Pearson’s correlations were calculated between EPO titres and mRNA expression of EPO, MYC and XBP1s mRNA expression to evaluate the relationship between these parameters. The threshold for statistical significance was *p* < 0.05.

## Supplementary Information

Supplementary Information.

## Data Availability

All the data generated and/or analysed during the current study are available in the article.
