# Supplementary material for: Engineering of Chinese hamster ovary cells for co-overexpressing MYC and XBP1s increased cell proliferation and recombinant EPO production
Source: Sci Rep. 2023 Jan 27;13:1482. doi: 10.1038/s41598-023-28622-z (PMC9883479; doi:10.1038/s41598-023-28622-z)
Supplement: Supplementary file 1 — Supplementary Information. [file 41598_2023_28622_MOESM1_ESM.pdf]

Research article

Engineering of Chinese hamster ovary cells for co-overexpressing MYC and XBP1s increased cell proliferation and recombinant EPO production

[Authors]

Correspondence: Prof. Claudia Altamirano, School of Biochemical Engineering, Pontifical Catholic University of Valparaiso, 2085 Brasil Av., Valparaiso, Chile.

E-mail: claudia.altamirano@pucv.cl

Keywords: Biopharmaceuticals, CHO cells, cell engineering, process engineering, protein secretion, cell proliferation.

Abstract

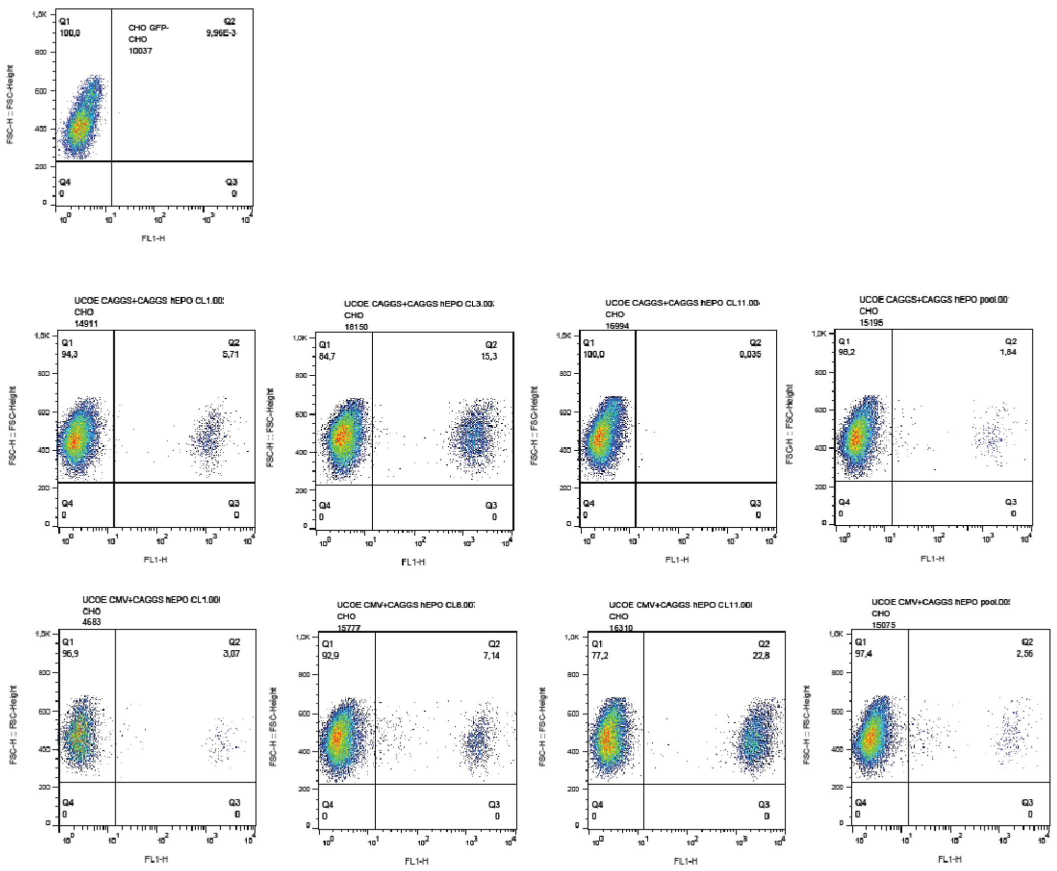

Figure S1. FACS analysis of selected clones after RMCE. X-axis corresponds to FSC. Y-axis correspond to FL1 channel (eGFP). At the top, the master cell line is found. At the bottom, the isolated 8 clones can be found.

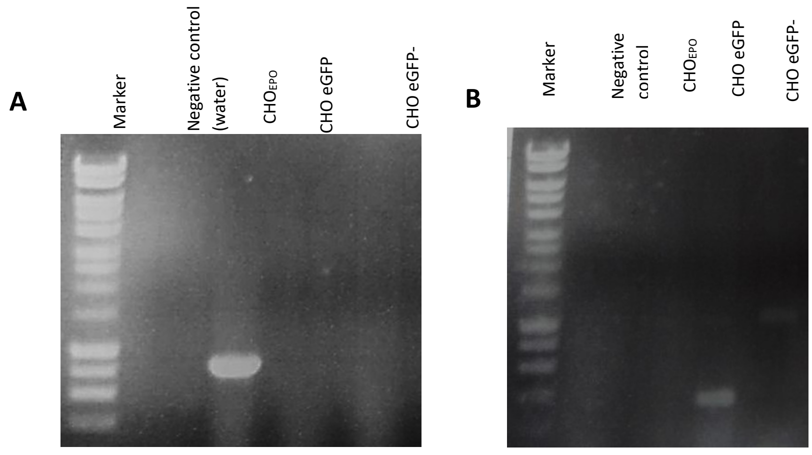

Figure S2. Analysis of the genomic DNA for EPO and GFP expression during the RMCE. A correspond to the EPO gene insetion in the EPO producing cell line. B correspond to the GFP insert in the master cell line.

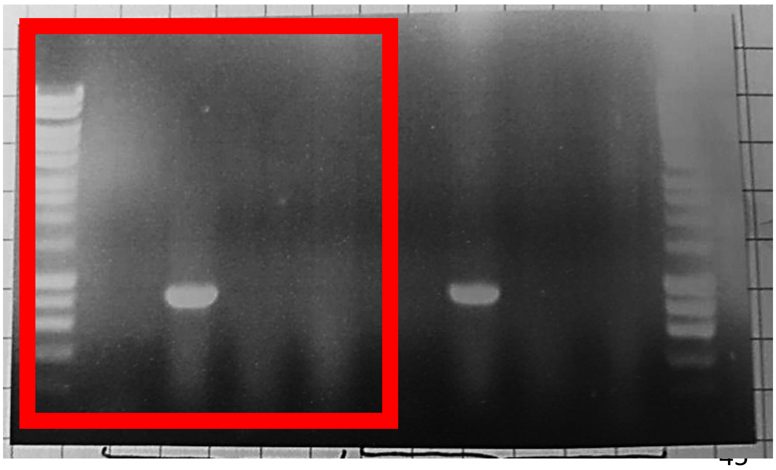

Left blot

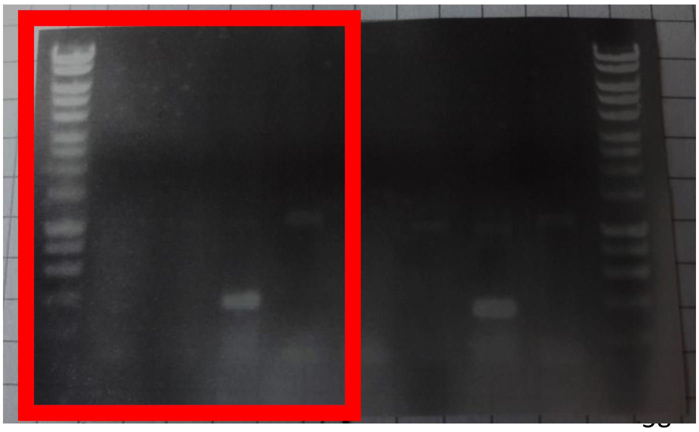

Right blot

Figure S3. Original blots for Figure S2.
